# Flow parsing and heading perception show similar dependence on quality and quantity of optic flow

**DOI:** 10.3389/fnbeh.2013.00049

**Published:** 2013-06-19

**Authors:** Andrew J. Foulkes, Simon K. Rushton, Paul A. Warren

**Affiliations:** ^1^School of Psychological Sciences, The University of ManchesterManchester, UK; ^2^School of Psychology, Cardiff UniversityCardiff, UK

**Keywords:** optic flow processing, heading, flow parsing, object movement, ego-motion

## Abstract

Here we examine the relationship between the perception of heading and flow parsing. In a companion study we have investigated the pattern of dependence of human heading estimation on the quantity (amount of dots per frame) and quality (amount of directional noise) of motion information in an optic flow field. In the present study we investigated whether the flow parsing mechanism, which is thought to aid in the assessment of scene-relative object movement during observer movement, exhibits a similar pattern of dependence on these stimulus manipulations. Finding that the pattern of flow parsing effects was similar to that observed for heading thresholds would provide some evidence that these two complementary roles for optic flow processing are reliant on the same, or similar, neural computation. We found that the pattern of flow parsing effects observed does indeed display a striking similarity to the heading thresholds. As with judgements of heading, there is a critical value of around 25 dots per frame; below this value flow parsing effects rapidly deteriorate and above this value flow parsing effects are stable [see Warren et al. (1988) for similar results for heading]. Also, as with judgements of heading, when there were 50 or more dots there was a systematic effect of noise on the magnitude of the flow parsing effect. These results are discussed in the context of different possible schemes of flow processing to support both heading and flow parsing mechanisms.

## Introduction

**Figure 1:**
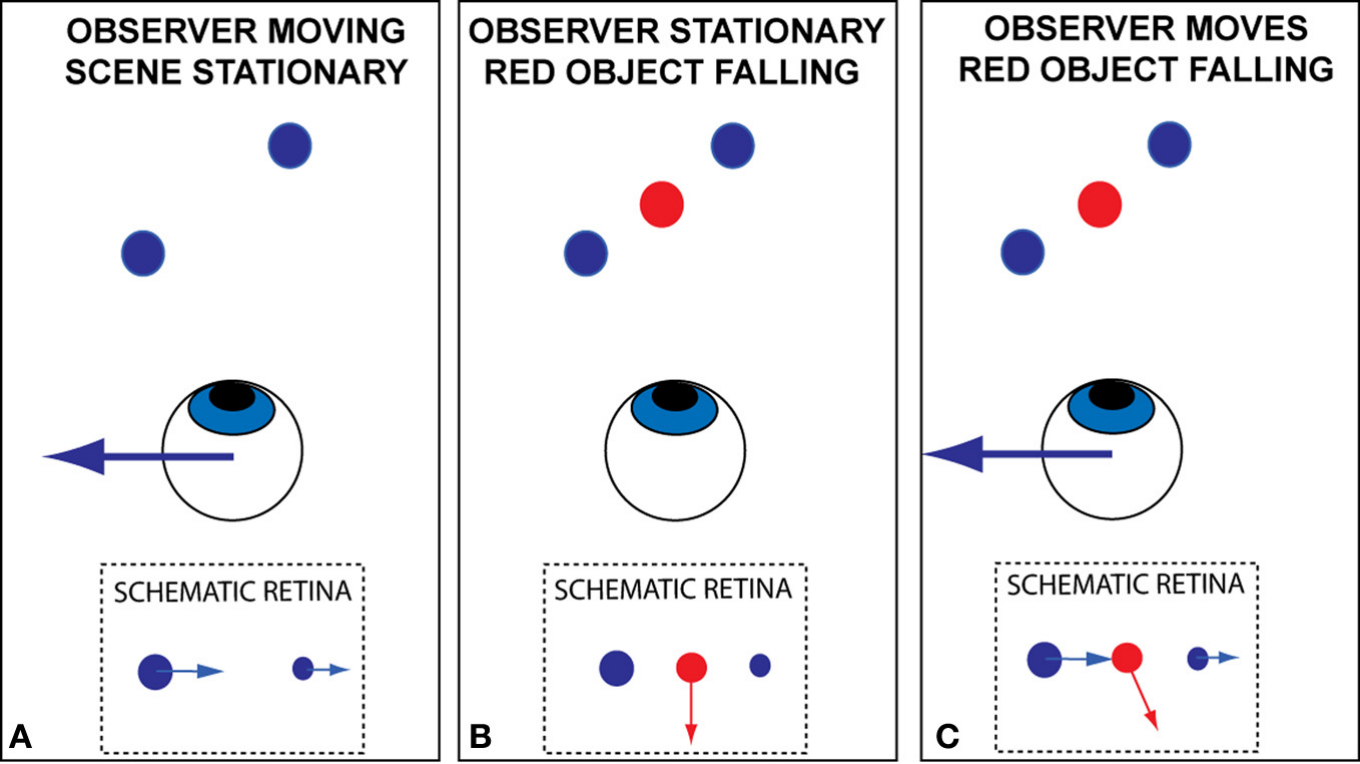
**Schematic illustration, in plan view, of the problem of recovering an appropriate estimate of scene-relative object movement when an observer is moving**. Retinal motion occurs when **(A)** the scene is stationary but the observer moves; **(B)** the observer is stationary but an object moves in the scene or **(C)** The observer moves and there are both stationary and moving objects in the scene.

Motion of the image of an object across the retina indicates relative movement between the object and the eye. The relative movement may have arisen due to movement of the object (Figure 1A), movement of the eye (Figure 1B), or a combination of the two (Figure 1C). How does the brain distinguish between these possibilities?

One solution is to use non-visual information such as copies of motor commands issued by the brain—efference copy, vestibular information, etc. If the observer moved then there should be a correlation between retinal and extra-retinal motion signals and the extra-retinal information can be used to compensate for the retinal consequence of the observer movement. The role of non-visual information in distinguishing between retinal movement due to the movement of the observer (“re-afference”) from retinal motion due to movement of objects in the environment (“ex-afference”) was described and investigated by von Holst (e.g., von Holst and Mittelstaedt, 1950) and explored further by Wallach (1987 for a review) and Gogel (1990 for a review).

Recently we have been investigating whether retinal information, specifically optic flow (the global patterns of retinal motion that are characteristic of self movement), can be used to distinguish retinal motion due to self-movement from retinal movement due to object movement (Rushton and Warren, 2005; Rushton et al., 2007; Warren and Rushton, 2007, 2008, 2009a,b; Warren et al., 2012).

The primate brain has a known neural sensitivity to optic flow (Duffy and Wurtz, 1991; Lappe et al., 1999; Morrone et al., 2000; Smith et al., 2006). Therefore, in principle it could identify global components of retinal motion that are likely due to self-movement and parse them out, isolating components of motion due to movement of objects within the scene. We have demonstrated that observers behave in a manner which is consistent with the existence of such a process (Rushton and Warren, 2005; Rushton et al., 2007; Warren and Rushton, 2007, 2009a). Others have characterized performance (Matsumiya and Ando, 2009; Royden and Connors, 2010; Calabro et al., 2011) and explored how non-visual information might contribute (Angelaki et al., 2011; Fajen and Matthis, 2011; MacNeilage et al., 2012). Recently we have begun exploring the mechanisms that underpin the “parsing” process (Warren and Rushton, 2009b; Warren et al., 2012).

There is a very extensive literature that shows observers can judge their instantaneous direction of movement (heading) from optic flow alone [for a review see Lappe et al. (1999) and see the companion paper to the present paper, Foulkes et al. (2013)]. In our experiments on flow parsing we have also found that observers can also judge scene-relative object movement in pure optic flow displays. An obvious question is whether both processes rely on the same neural processing mechanisms. One way to explore this question is to look for signatures of common processing.

In the companion paper to the present paper (Foulkes et al., 2013) we examined how the precision and accuracy of human heading judgements varied as a function of the quality (noise in the flow field) and quantity (number of flow vectors present) of the optic flow to generate a performance profile. We then compared the profile to similar performance profiles (accuracy and precision as a function of the quality and quantity of optic flow) for four candidate models of human heading computation. Here we derive a similar characterization of “flow parsing” as a function of the quality and quantity of optic flow available. This allows us to see whether the performance profiles are similar in the two tasks—if they are then this lends weight to the conclusion that they share neural processing mechanisms.

In the heading judgement tasks, thresholds for human performance decreased as the quantity of dots increased from 5 to 25–35 optic flow vectors. Beyond this point thresholds were relatively stable irrespective of the quantity of flow vectors. This data is comparable to that found in Warren et al. (1988), which also examined the effects of flow field density on heading thresholds. Also beyond 25–30 dots, heading thresholds increased as a function of directional noise in the flow field. In the present experiment we will assess whether a similar profile arises for a flow parsing task.

The approach used in our previous flow parsing studies has involved participants making judgements about the perceived trajectory of an onscreen probe object that was contralateral to a hemi-field of optic flow (Warren and Rushton, 2009b). We have argued that if observers are able to perform something akin to a subtraction of a global component of flow associated with self-movement then the presence of optic flow should have a predictable effect on the perceived trajectory of the probe. Specifically, when the optic flow is an expanding radial flow field, due to global subtraction of the outwards field, the perceived probe trajectory should be biased inwards toward the focus of expansion. Here, we rely on a similar rationale and experimental paradigm in order to assess how flow parsing varies as a function of the quantity and the quality of information in the flow field.

To pre-empt the results we find that the profile for the parsing effect is very similar to that seen for the heading estimation task. Specifically there is a significant reduction in the effect when the brain has access to fewer than 25 dots per frame but beyond this quantity of flow vectors the effect is relatively stable. In addition, when there are more than 25 dots per frame the magnitude of the effect depends upon the quality of the flow information, with increasing levels of noise leading to smaller effects.

## General methods

### Participants

Twelve observers took part in experiment 1 and eleven observers took part in experiment 2. All participants worked or studied in the School of Psychological Sciences, University of Manchester. Two authors participated in both studies (Paul A. Warren and Andrew J. Foulkes), all other participants were naive regarding the purpose of the study. All participants had normal or corrected to normal vision. Recruitment and testing procedures were in line with the Declaration of Helsinki and were approved by the appropriate institutional ethics committees.

### Apparatus

Observers were seated with the chin positioned in a chin rest in a dark room with the eyes at a distance of approximately 57 cm from the display. Stimuli were presented on a 22″ Viewsonic (pf225) CRT with 100 Hz frame rate and resolution 1024 × 768 pixels. The visible portion of the CRT subtended ~40 × 30°. To minimize stray light, irregularly shaped black card was used to obscure the CRT casing and the edges of the visible portion of the screen. We used Lazarus (a free, open source development system for Pascal—http://www.lazarus.freepascal.org) together with the JediSDL libraries (http://jedi-sdl.pascalgamedevelopment.com/) to code the experiments. The displays were rendered using OpenGL on a NVidia GeForce 9600GT 512Mb graphics card with 16x anti-aliasing.

### Stimuli

Stimuli were onscreen for 2 s and comprised a moving cloud of red limited lifetime (25 frames = 250 ms) dots on a black background. The field of dot motion simulated forward observer movement at a speed of 1 m/s, with the median onscreen dot speed ~5.4°/s. The focus of expansion of the resultant radial flow field was coincident with the center of the screen.

At generation and regeneration (i.e., when lifetime had expired), 2D on-screen dot location was sampled randomly from a uniform distribution and then assigned a random depth in the range 0.5–4.5 m from the observer before perspective back-projection to obtain a 3D location.

**Figure 2:**
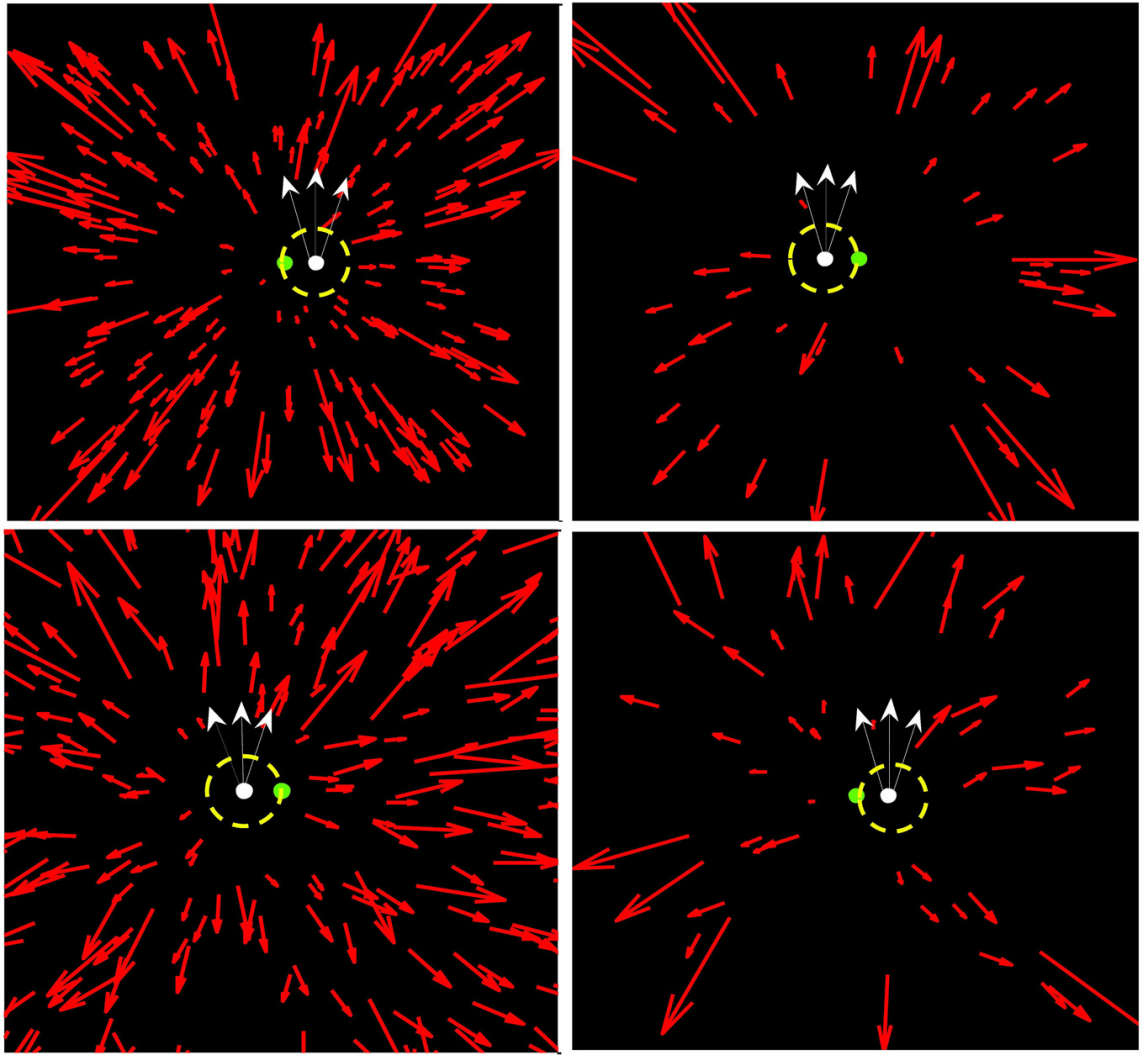
**Sample stimuli: (Top left): 200 points, no noise; (top right) 50 points, no noise; (bottom left) 200 points, 15° s.d. noise; (bottom right) 50 points, 15° s.d. noise**. We also show the fixation point (in green) with white probe moving in one of three possible directions starting at one of two locations. The dashed circle is a 3° aperture which the radial flow dots could not enter. This minimized the contribution of local motion processing mechanisms.

We manipulated the number of dots in the flow field on a given frame (see Figure 2). We also varied the directional noise in the vectors comprising the flow field. Specifically, at the point of generation the direction of each vector in the flow field was corrupted by independent, additive zero mean Gaussian noise, such that for an optic flow vector ***v*** = (*r*, θ)^*T*^ in the radial flow field, the associated vector in the noisy flow field was given by ***v***′ = (*r*, θ + *N*(0, σ))^*T*^.

In addition, to the dots in the flow field, a small (0.09° diameter), white probe dot was present in the display (Figure 2). The dot moved at a speed of ~0.6°/s on a linear trajectory either to the left or right of fixation. In order to minimize the contribution of local motion processing mechanisms to the effects seen, the probe was surrounded by a black circular mask of radius 3° which obscured the flow information neighboring the probe (see Warren and Rushton, 2009b).

In all trials a green fixation spot was presented in the middle of the screen, which coincided with the focus of expansion of the radial flow field (Figure 2).

## Experiment 1

In the first experiment, we quantified the effects of changes in the amount of directional noise and number of flow vectors on flow parsing. As explained in the Introduction, if flow parsing and heading estimation are underpinned by common neural processing we would expect to find a similar pattern in the flow parsing effect data to that seen in the heading threshold data in Foulkes et al. (2013).

### Design

In experiment 1 we manipulated quality (amount of noise) and quantity (number of flow vectors) of the flow field for two probe positions and three probe movement directions.

The quantity of flow field information was manipulated by varying the number of dots present per frame (5, 50, 100, 200 dots/frame). It should be noted that given the limited lifetime of our dots (250 ms) and an estimate of visual persistence of around 100 ms (see Di Lollo, 1980) we expect that the brain actually has access to ~40% more points than are present on any single frame.

The quality of the flow field information was controlled by corrupting each flow vector direction with an independent additive Gaussian noise process (see stimulus section). To vary the amount of noise we adjusted the standard deviation of the Gaussian noise process (0, 7.5, 15°).

The quality and quantity factors were the two experimental factors of interest. Probe position and movement direction were manipulated simply to improve data quality. The probe moved in one of three directions (75, 90, 105°), centred on the vertical upwards direction (90°). The reason for this manipulation was to ensure that participants never knew whether perceived motion was real, illusory, or a combination of the two. Finally, so that participant responses were not always in one direction the start point of the probe motion was either presented 3° to the left or 3° to the right of fixation. The data were processed such that we were able to average responses over the probe direction and position factors (See Data processing and analysis section).

**Figure 3:**
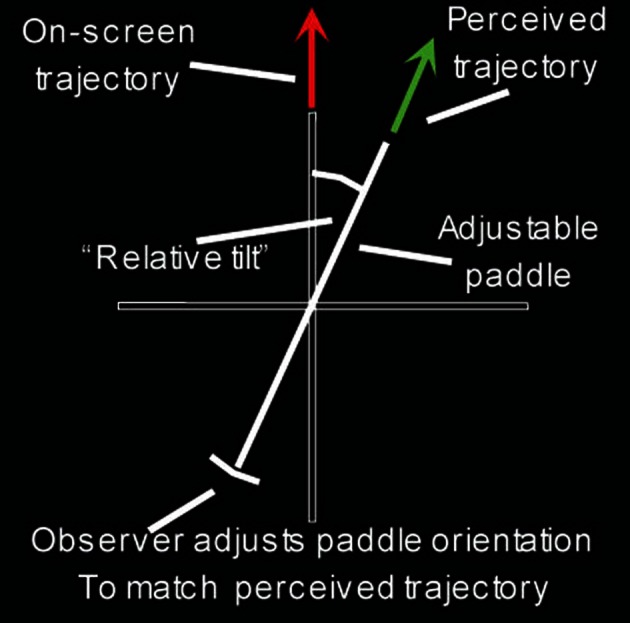
**The adjustable onscreen gauge [reproduced from Warren et al. (2012)]**. The red arrow represents the actual trajectory of the probe, with the green arrow the observer's perceived probe direction.

Participants indicated the perceived direction of movement of the probe (see procedure section). We call the difference between the perceived and physical trajectories the “relative tilt” (Warren and Rushton, 2009a,b; see Figure 3). The magnitude of the relative tilt is a measure of the effect of the global subtraction process. Participants saw each condition (flow quantity, flow quality, probe position, probe direction) twice per session, giving 144 trials in total in each of two sessions.

### Procedure

In each trial, initially a red fixation cross appeared for 1 s to give the participant a warning that the trial was about to start. The red cross turned into a green annulus and the stimulus was presented. Observers then indicated the perceived trajectory of the probe by using a mouse to rotate an onscreen paddle. The paddle was drawn against reference horizontal and vertical axes (Figure 3).

Participants were instructed to adjust the orientation of the paddle until it matched the perceived orientation of the trajectory of the probe dot, relative to where the dot first started. Participants were told that if the probe was seen to move along a curved trajectory they should set the paddle to a straight line fit to the perceived trajectory.

### Data processing and analysis

As explained in the design section, the dependent variable, relative tilt, was defined as the difference between the perceived and onscreen probe trajectories. The coordinate system and sign convention were defined such that 0° corresponded to the positive x-axis and angles increased in the anticlockwise direction. To make the data consistent across probe direction conditions, for each trial relative tilts obtained in the 75 and 105° probe angle conditions (i.e., 15° either side of vertical) were transformed to the equivalent quantity, ${\hat{\theta }}_{\text{RT}}$, which would have been obtained if the probe had moved vertically using Equation 1, in which θ_RT_ and θ_P_ correspond to the relative tilt measured and the probe angle (see Warren and Rushton, 2007 for a derivation):

(1) $${\hat{\theta }}_{RT}=\tan^{-1}\left(\sin\theta_{RT}/\sin\theta_{P}\right),$$

Perceived trajectory was then averaged over probe angle conditions. The sign of the data was flipped for the −3° probe conditions and combined with the +3° data.

In previous experiments, although the pattern of results was consistent across participants there was variability in the magnitude of the effect. To minimize this source of variability we normalized relative tilt scores as follows. After taking averages in relative tilt over repetitions, probe positions and probe directions we calculated the grand mean for each participant over the different quality and quantity conditions. Each relative tilt data point was then divided by the grand mean so that it now characterized the proportional size of the relative tilt effect in that condition compared to the grand mean over all conditions.

### Results

**Figure 4:**
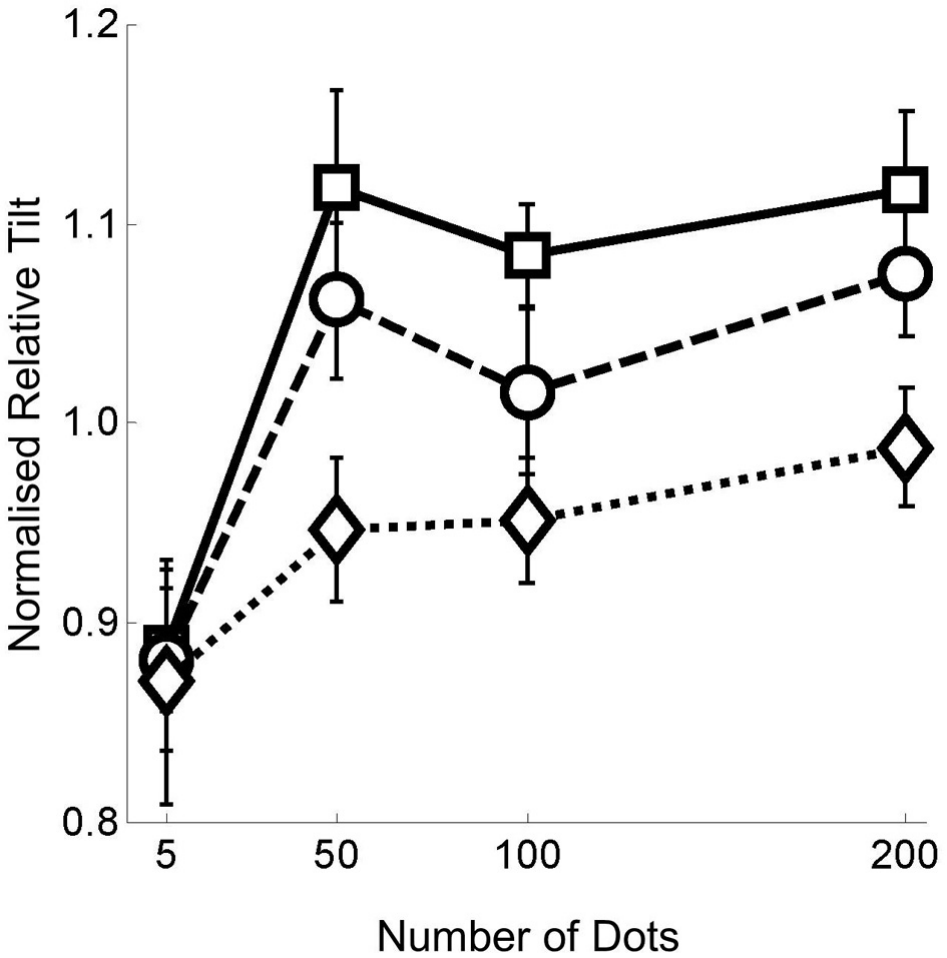
**Data from experiment 1 with number of dots per frame on the horizontal axis, and normalized relative tilt on the vertical axis**. The three different plots on each graph represent the data for the three noise levels—squares with solid lines for no noise; circles with dashed lines for noise level 1; and diamonds with dotted lines for noise level 2. Error bars represent standard errors.

Figure 4 shows the normalized mean relative tilt data across all 12 participants for the flow quantity range 5–200 dots per frame. We show the relative tilt data as a function of the flow quantity factor for each of the flow quality conditions. In order to give an indication of the magnitude of the relative tilt effect before normalization the grand mean of the effect over all conditions and observers was ~20°.

First, it can be seen that there is a significant reduction in the flow parsing effect with only five dots in the flow field. Second, the magnitude of the effect appears to plateau once there are 50 or more dots. Third, once there are 50 or more dots, a clear difference between the noise levels is apparent. The first of these observations was tested by conducting a 2 × 3 repeated measures ANOVA on the 5 and 50 dots per frame data across all three noise levels. The ANOVA revealed only an interaction between the quantity and quality manipulations [*F*_(2, 22)_ = 6.068, *p* = 0.008] due to the fact that the effects observed were similar at 5 but not 50 points per frame. The second and third observations were tested by conducting a 3 × 3 repeated measures ANOVA on the 50, 100, 200 dots per frame data across all three noise levels, revealing only a main effect of the flow quality manipulation [*F*_(2, 22)_ = 10.426, *p* = 0.001]. We then performed 1 factor ANOVAs at each of quantity levels which revealed a significant effect of flow quality at every level except five points per frame [five points: *F*_(2, 22)_ = 0.052, *p* = 0.949; 50 points: *F*_(2, 22)_ = 4.730, *p* = 0.020; 100 points: *F*_(2, 22)_ = 3.695, *p* = 0.041; 200 points: *F*_(2, 22)_ = 5.306, *p* = 0.013]. These analyses are presented in greater detail in the appendix.

## Experiment 2

### Rationale

It is apparent from the last experiment that the critical range for performance is between 5 and 50 dots per frame. Consequently in the second experiment we examined flow parsing effects in this range in more detail.

### Design

The design of experiment 2 was exactly the same as experiment 1. All experimental factors remained the same save the levels of the flow quantity factor which were set at 5, 15, 25, 35, and 50 dots per frame.

### Procedure, data processing, and analysis

The procedure used was exactly the same as that described in experiment 1, as was the data processing and analysis.

### Results

**Figure 5:**
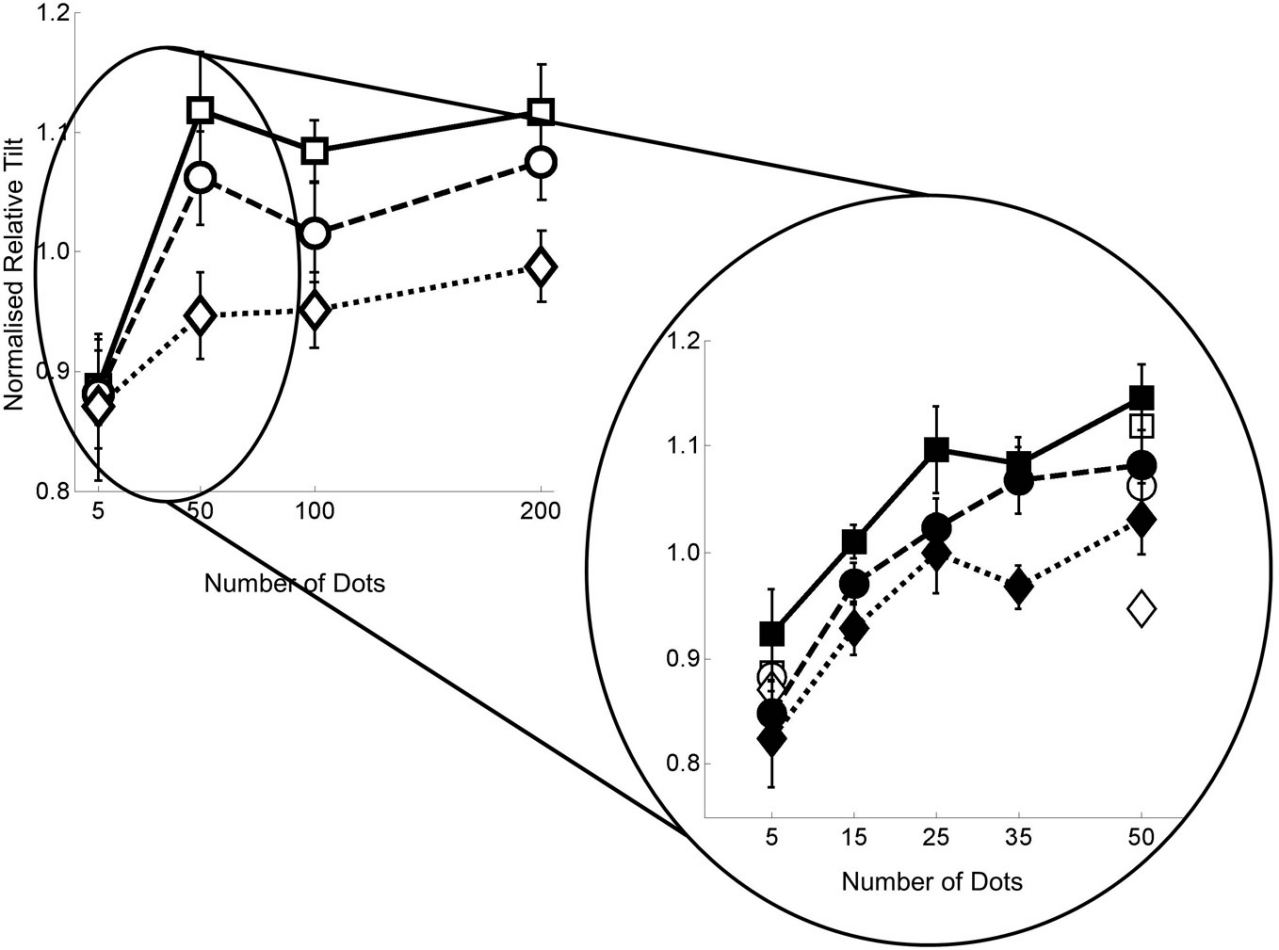
**Data from experiment 2 with number of dots per frame on the horizontal axis, and normalized relative tilt on the vertical axis**. The three different plots on each graph represent the data for the three noise levels—squares with solid lines for no noise; circles with dashed lines for noise level 1; and diamonds with dotted lines for noise level 2. Filled shapes represent the 5–50 data and hollow shapes the 5–200 data. Error bars represent standard errors.

Figure 5 shows the normalized mean relative tilt across all 11 participants for the dots per frame range 5–50 as an insert into the data already presented in Figure 4. In order to give an indication of the magnitude of the relative tilt effect before normalization in experiment 2 the grand mean of the effect over all conditions and observers was ~14°.

It can be seen from the figure that the relative tilt effect stabilizes at around 25 points per frame. To test this assertion we ran four 2 × 3 repeated measures ANOVAs (one for each of the neighboring flow quality pairs, i.e., 5–15, 15–25, 25–35, 35–50) to determine the ranges for which there was no main effect of number of points. We confirmed that the 25–35 range was the first range for which there was no main effect of number of dots per frame [*F*_(1, 10)_ = 0.001, *p* = 0.980]. See Appendix for the details of this analysis. We note that relative to experiment 1 there appears to be a greater effect of the noise manipulation in the lowest dots per frame condition in this experiment. However, we then investigated the point at which the addition of noise led to a difference in the relative tilts by running a series of 5 one factor repeated measures ANOVAs—one at each flow quantity level. In partial agreement with experiment 1, we found that there was only marginal evidence for a main effect of the noise manipulation at the five dots per frame level [*F*_(2, 20)_ = 3.222, *p* = 0.061]. However, by 35 dots per frame the effect of the noise manipulation was highly significant [*F*_(2, 20)_ = 6.58, *p* = 0.006). See Appendix for details of this analysis.

### Comparison with heading data

As noted in the Introduction, we have also collected data on a heading task using a similar design and similar stimuli (Foulkes et al., 2013). Now we examine the performance profiles for the two tasks. Similarities would support the hypothesis that the flow parsing and heading estimation mechanisms share some neural processing.

**Figure 6:**
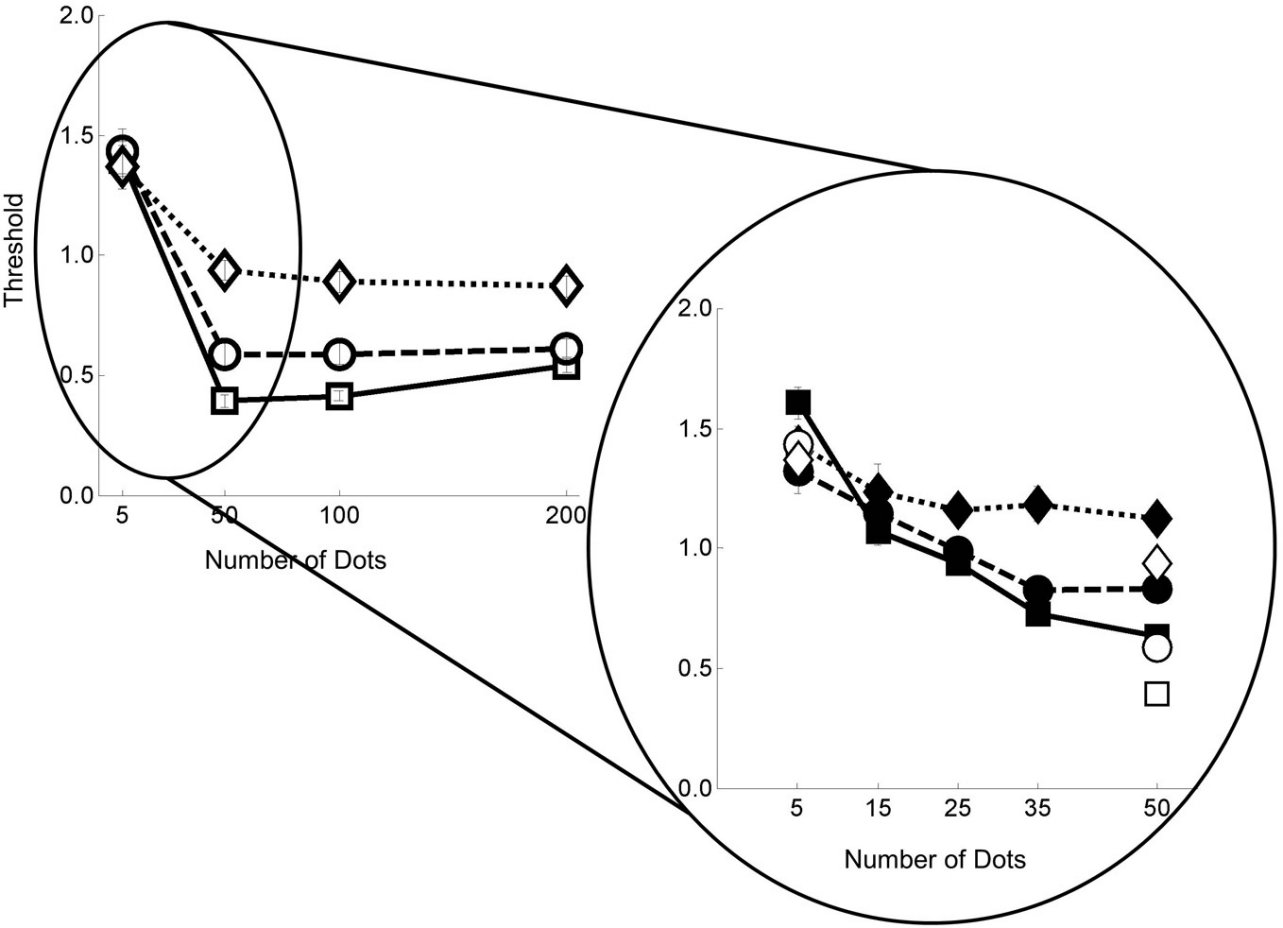
**Normalized weighted thresholds for the twelve flow field conditions**. As with the flow parsing data, squares with solid lines represent no noise, circles with dashed lines for noise level 1, and diamonds with dotted lines for noise level 2. Filled shapes are for the 5–50 data and hollow shapes for 5–200. Error bars represent ±1 s.e.

In Figure 6 we re-plot the heading data from Foulkes et al. (2013) for comparison with the data in Figure 5. We point to four features in common for these two data sets. First with five dots per frame, there is little difference between the noise conditions. Second with 50 or more dots a performance plateau is reached. Third, with 50 or more dots there is a clear separation between noise conditions. Finally, for both parsing and heading tasks, when we look closer at the region between 5 and 50 dots per frame we find that the critical range is around 25–35 dots per frame—below that there is a rapid change [again this value is in line with previous data describing the relationship between heading thresholds and dot density found in Warren et al. (1988)].

**Figure 7:**
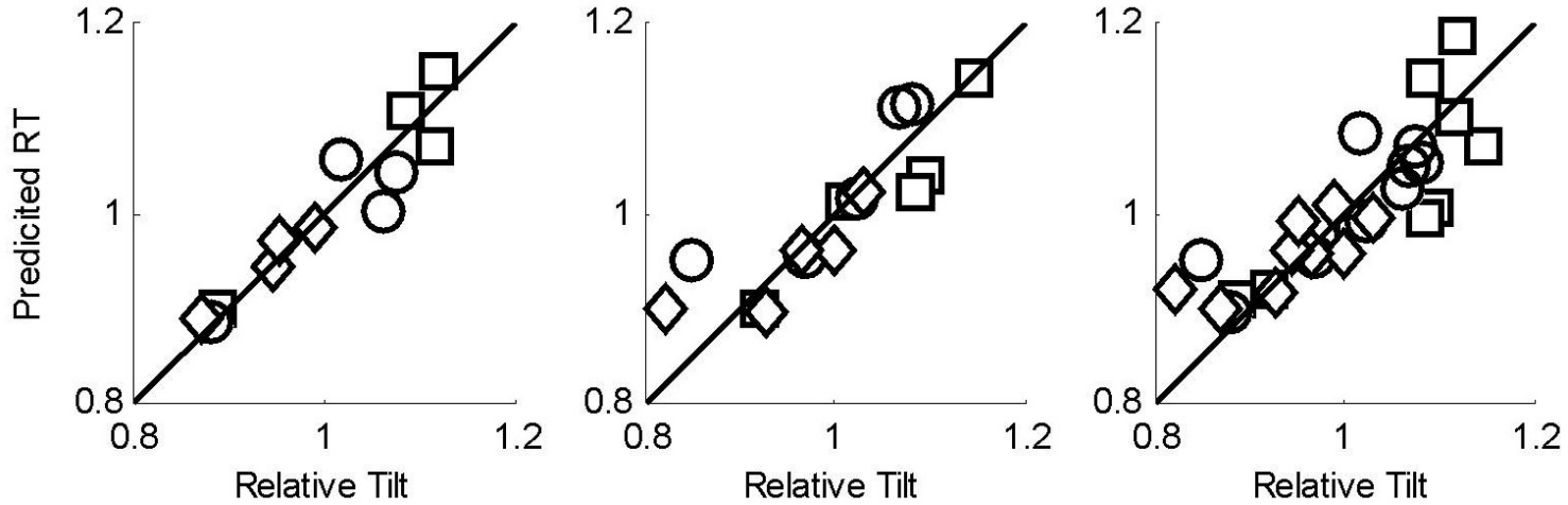
**Results of regression analysis to predict the relative tilt data from the sensitivity data obtained in heading task**. Squares represent the no noise data, circles noise level 1, and diamonds noise level 2. **(left)** 5–200 points data, **(center)** 5–50 points data; **(right)** all data. The solid line is the line Predicted Relative Tilt = Relative Tilt.

In order to compare formally the data in the two tasks, we first converted thresholds to sensitivities (reciprocal of the threshold). We then conducted a linear regression in which the relative tilt was regressed against the heading sensitivity measure. In Figure 7 we show the outcome of this analysis.

We see from Figure 7 (left panel) that for the 5–200 data, there is a strong relationship between the heading sensitivity and the relative tilt data (*p* = < 0.001, *R*^2^ = 0.88). For the 5–50 data, we see that the relationship is also strong (*p* = < 0.001, *R*^2^ = 0.74), as is the relationship when all the data from experiments 1 and 2 are combined and regressed against the commensurate heading threshold data (*p* = < 0.001, *R*^2^ = 0.69). We show the full details of the analysis in the appendix.

## Discussion

### Summary

We have investigated the dependence of flow parsing on both the quantity (number of flow vectors in the image) and quality (amount of noise in the flow vector directions) of optic flow information. We found that there appears to be a critical level of flow quantity (around 25 dots per frame) above and below which the patterns of relative tilt effects observed are qualitatively different. Below the critical level the relative tilt effect depends on the flow quantity, decreasing as fewer dots are presented. In addition below the critical level the effect of flow quality on flow parsing is seen to diminish with no difference in the size of the effect between the different noise levels at the lowest value of flow quantity tested. Above the critical level the pattern of effects is quite different. The relative tilt effect no longer appears to depend on the flow quantity but does depend on the flow quality, such that as directional noise is added the effect is reduced. The pattern of effects is broadly consistent with those observed in a related study on heading estimation using a similar optic flow stimulus.

### Heading and flow parsing—served by common flow processing?

We designed an experiment to examine whether heading and parsing show similar performance profiles. They do, which suggests that heading estimation and flow parsing rely on common processing to estimate self-movement. However, it is worth noting that work we have described elsewhere indicates that parsing does not rely on a prior estimate of heading (Warren et al., 2012). Therefore, we are left with two alternatives to consider. Either heading or parsing both rely on a common early stage in which direction of self-movement is estimated such that the output of one mechanism does not provide input for the other, or heading relies on the output of the parsing system. In future work we aim to distinguish between these two possibilities.

### Optic flow processing mechanisms

The performance of participants in both tasks in the face of our noise and density manipulations has clear implications for optic flow processing mechanisms that might underpin both flow parsing and heading recovery. The sharp improvement in performance up to around 25–35 dots per frame and drop off in performance improvement after that suggests that even though the task is still possible for the most sparse case tested here, a minimum number of image points is required to support robust performance. This finding is in line with previous studies on heading perception suggesting an ~N^−0.5^ relationship between thresholds and density (Warren et al., 1988). When taken together with the relative robustness to noise exhibited in both tasks, we suggest that our results point strongly toward a shared global motion processing stage that integrates motion information over the visual field (which may implicate cells in area MST). These findings are also consistent with our own previous studies on flow parsing which emphasize the importance of global motion processing (Warren and Rushton, 2009a,b).

### Robustness of flow parsing

It is interesting to consider the robustness of the flow parsing mechanism to changes in the quality and quantity of information in the flow field. Although there was a (statistically) significant reduction in the magnitude of the effect when the stimulus was composed of five dots per frame, the relative tilt magnitude was still 80–95% (depending on the flow quality condition) of that seen when there were 250 dots per frame. Similarly, even in the conditions in which the noise manipulation had the biggest effect (i.e., when the flow field contained many dots), the relative tilt magnitude in the highest noise condition was still on the order of 85–90% of that seen when there was no noise.

This robustness is reassuring given that the visual system sometimes faces conditions in which there is reduced visual information (e.g.. in fog, sparse environments). In addition, as well as any sensory noise in the representation of motion information, the visual system must also cope with ambiguity in input motion signals due to the aperture problem (Nakayama and Silverman, 1988a,b). The aperture problem occurs because the primary motion sensors in the visual system have local receptive fields and consequently cannot uniquely specify motion of relatively larger objects. This ambiguity is equivalent to an additional source of noise in the input motion vectors. Since the visual system is exposed to low density conditions and must deal with input noise, a mechanism which is responsible for assessing scene-relative object motion from visual self-movement information (or estimating heading) should be able to operate under such circumstances and our data confirm that this is the case for flow parsing.

### Noise manipulation

We used noise to derive a “signature” of human performance that could be compared across tasks (flow parsing and heading in this paper) and in the companion paper across models. The choice of the type of noise was somewhat arbitrary although informed by the previous literature (Warren et al., 1991). We used an unbiased Guassian noise process to perturb motion direction. Alternatives might have been the selective perturbation of speed, the combined perturbation of speed and direction, the addition of outlier noise, or temporal noise (manipulating the lifetime of the dots to change the ratio of signal to noise dots).

## Conclusions

The data presented in these experiments suggest that there are a number of similarities between the performance of the flow parsing and heading estimation mechanisms. Both exhibit a similar pattern of dependence on—but also appear relatively robust to changes in—the quality and quantity of information in the optic flow field. These findings suggest that they share common neural mechanisms for assessment of observer movement and that these involve global motion processing.

### Conflict of interest statement

The authors declare that the research was conducted in the absence of any commercial or financial relationships that could be construed as a potential conflict of interest.

## Appendix: statistical analyses

In this appendix, we provide further details of the data analysis of the two experiments in the main article.

### Experiment 1

We present the results of several repeated measures ANOVAs conducted on the data for experiment 1. First we conducted a 3 × 3 ANOVA on the 50, 100, 200 dots per frame conditions across all three noise conditions. The results are shown in Table A1 and suggest that there is no effect of the flow quantity manipulation beyond the 50 dots per frame level. There is, however, a clear effect of the flow quality manipulation.

**Table A1:** **Outcome of 3 × 3 Repeated Measures ANOVA tests of the effects of flow quality and quantity for the 50, 100, 200 number of points levels across all three noise conditions**.

**Factor**	***F***	***p***
Number of points	*F*_(2, 22)_ = 1.005	0.382
Noise	*F*_(2, 22)_ = 10.426	0.001
Interaction	*F*_(2, 22)_ = 0.221	0.925

We also conducted three 2 × 3 ANOVAs on the data for each successive pair of number of points per frame conditions across all three noise levels. The outcome is presented in Table A2. Note that the number of points factor is only significant for the 5–50 comparison. In addition there was a significant interaction present for this comparison which was driven by the fact that that there was no difference in relative tilt at five points per frame but a significant difference at 50 points per frame (see Table A3). In agreement with the ANOVA conducted in Table A1 we see for the other number of points comparisons (50–100 and 100–200) there were only main effects of the flow quality manipulation.

**Table A2:** **Results of 2 × 3 Repeated Measures ANOVA tests of the effects of flow quantity and quality at each consecutive pair of points per frame level**.

**Number of points**		***F***	***p***
5, 50	Points	*F*_(1, 11)_ = 10.038	0.009
	Noise	*F*_(2, 22)_ = 1.870	0.178
	Interaction	*F*_(2, 22)_ = 6.068	0.008
50, 100	Points	*F*_(1, 11)_ = 0.580	0.462
	Noise	*F*_(2, 22)_ = 8.055	0.002
	Interaction	*F*_(2, 22)_ = 0.259	0.774
100, 200	Points	*F*_(1, 11)_ = 2.358	0.153
	Noise	*F*_(2, 22)_ = 9.204	0.001
	Interaction	*F*_(2, 22)_ = 0.097	0.908

**Table A3:** **Results of One-Way Repeated Measures ANOVA tests of the effect of flow quality at each level of the number of points per frame factor**.

**Number of points**	***F***	***p***
5	*F*_(2, 22)_ = 0.052	0.949
50	*F*_(2, 22)_ = 4.730	0.020
100	*F*_(2, 22)_ = 3.695	0.041
200	*F*_(2, 22)_ = 5.306	0.013

Finally, we conducted four One-Way ANOVAs at each level of the number of dots per frame factor across the three noise levels. We found no effect of noise for five points per frame, but a main effect in all three other cases. We show the results in Table A3.

### Experiment 2

In Table A4 we present the results of four 2 × 3 RM ANOVA tests for neighbouring dot conditions across all three noise conditions. We found main effects of the number of dots per frame factor for the 5–15 and 15–25 cases, but not for the 25–35 and 35–50 cases. This suggests that beyond 25–35 dots per frame the relative tilt effect is stable. In contrast the noise factor was significant across all number of points comparisons.

**Table A4:** **Results of 2 × 3 Repeated Measures ANOVA tests of the effects of flow quantity and quality at each consecutive pair of points per frame level**.

**Number of points**		***F***	***P***
5, 15	Points	*F*_(1, 10)_ = 10.175	0.001
	Noise	*F*_(2, 20)_ = 6.102	0.009
	Interaction	*F*_(2, 20)_ = 0.304	0.742
15, 25	Points	*F*_(1, 10)_ = 19.519	0.001
	Noise	*F*_(2, 20)_ = 3.718	0.042
	Interaction	*F*_(2, 20)_ = 0.148	0.864
25, 35	Points	*F*_(1, 10)_ = 0.001	0.980
	Noise	*F*_(2, 20)_ = 4.674	0.022
	Interaction	*F*_(2, 20)_ = 0.715	0.501
35, 50	Points	*F*_(1, 10)_ = 1.878	0.201
	Noise	*F*_(2, 20)_ = 14.490	<0.001
	Interaction	*F*_(2, 20)_ = 0.297	0.746

Finally, in Table A5 we present the results of five One-Way ANOVAs conducted for each dots per frame condition across all three noise levels.

**Table A5:** **Results of One-Way Repeated Measures ANOVA tests of the effect of flow quality at each level of the number of points per frame factor**.

**Number of points**	***F***	***p***
5	*F*_(2, 20)_ = 3.222	0.061
15	*F*_(2, 20)_ = 4.752	0.020
25	*F*_(2, 20)_ = 2.427	0.150
35	*F*_(2, 20)_ = 6.580	0.006
50	*F*_(2, 20)_ = 16.135	0.002

We found that there was an effect of the noise manipulation at all levels except 5 and 25 dots per frame. We might have expected there to be a critical level of the number of dots per frame factor beyond which the noise manipulation began to have an effect. This is not the case in our analysis. However, we suspect that this is simply due to sampling error and that with more data the critical value would have been around the 25–35 dots per frame level.

### Regression analysis

We saw in the main article that there were similarities between the patterns of data for the relative tilt data from the flow parsing task and the heading threshold data obtained in a comparable heading task. To assess the strength of this relationship we performed regressed the flow parsing and heading data. We fit a linear model of the form

$$\tau_{F}=\alpha +\beta \tau_{S},$$

where τ_*F*_ and τ_*S*_ refer to the relative tilt data heading threshold data respectively. We undertook three separate regression analyses for the 5–200 data, the 5–50 data and the combined data set. We show the results in Figure 7 and also in Table A6 below.

**Table A6:** **Results of the regression analyses**.

	**α**	**β**	***F***	***p***	***R*^**2**^**
Experiment 1	0.799	0.148	*F*_(1, 10)_ = 76.267	<0.001	0.88
Experiment 2	0.707	0.264	*F*_(1, 13)_ = 37.866	<0.001	0.74
Combined	0.801	0.164	*F*_(1, 25)_ = 55.659	<0.001	0.69

It is clear that the fits were highly significant for all three cases suggesting that the heading and flow parsing data are strongly correlated and thereby providing evidence that they are underpinned by similar neural processing.
